# Carcinoma Uteri with Pregnancy

**Published:** 1854-10

**Authors:** John G. House

**Affiliations:** Springville, N. Y.


ART. IV.—*Carcinoma Uteri with Pregnancy.* By JOHN G. HOUSE, M. D., Springville, N. Y.

Carcinoma uteri, conjoined with gestation, is one of those combinations occurring so rarely, that I have deemed the following case worthy of a place on record. I will transcribe from my *case-book.*

Mrs. E., the subject of this disease, 40 years of age, the mother of six children, is now (April 17th) pregnant at the sixth month; has had two attacks of hemorrhage, the second of which was profuse. The os tincæ and cervix large and ragged. I entertain fears of placenta prævia.

℞ Plumbi acet., gr. ii.

Morph. sulph. gr. $\frac{1}{4}$, *pro re nata.*

Rest and cool regimen.

May 17th. Mrs. E. has had another attack of hemorrhage. My suspicions are more confirmed by all the symptoms. I think I can recognize the rugose surface of the placenta through the dilated os uteri.

℞ Same as above.

June 14th. The patient in whom I feared placenta prævia has this time pains without hemorrhage. The uterus, upon examination, feels hard and rigid like scirrus, occupying nearly the whole of the pelvis in its lateral expansion. The os itself is ragged and thickened. She has a very fetid and purulent discharge per vag. I am compelled to abandon my former suspicions, and pronounce the disease *Carcinoma Uteri.*

There is no doubt that she is pregnant, at 8½ months, for she says she feels motion, and the fœtal heart can be distinctly heard in the right hypogastrium.

℞ Morph. Sulph. gr $\frac{1}{4}$, *pro re.*

June 16. Continues in pain.

℞ Cathartic followed by morphine.

June 19th. Same as when last seen. Examined by my friend, Dr. Emmons, who pronounces the disease cancerous. We have frankly stated to her friends the probable unfavorable termination of the case.

July 1. Mrs. E. was yesterday visited by a German practitioner, who declared the doctors entirely mistaken, and the disease to be *Polypus Uteri*. He attempted its extraction, but, at length, by the good sense and firmness of some of the women in attendance, was forced to desist, and convinced of his error. Being thus thoroughly vanquished, he soon left in confusion, amid a shower of maledictions. The truth is, she is pregnant at full time, and has carcinoma uteri — a bad complication. There is now no evidence of life in the fœtus, although yesterday, I understand, the motion was strong.

6th. Has sharp, cutting pains, without any dilatation of the os uteri.

℞ Morph. *pro re*, which brings relief.

7th. More comfortable. Continue the treatment.

8th. Mrs. E. was visited to-day by Dr. Emmons, who coincides with me in diagnosis and treatment.

℞ Starch and Laud. enemata.

9th. More comfortable under the influence of the enemata.

10th. Patient is very sick; has chills and fever. The uterus is probably inflamed just above the cervix; she is helpless, and cries out on the slightest pressure; has a sanious discharge, but no dilatation; pulse 145.

℞ Wine negus and Dover's powder, as palliatives.

The case is considered hopeless.

11th. Has some hemorrhage; cold extremities; no dilatation; pulse 140, and supposed to be dying.

12th. Mrs. E. died to-day at 5 o'clock P. M. The friends are clamorous for an examination. I shall gratify their wishes and mine to-morrow morning.

13th. *Autopsy.* A crucial incision of the abdomen revealed a full-grown fœtus. The presentation the 2d of Baudelocque, this accounting for the sound of the fœtal heart being so distinctly heard in the right hypogastrium. Placenta attached to the posterior surface of the uterus, and rather smaller than usual. It separated easily, beautifully exhibiting its vascular connection. Uterus appeared healthy. The cervix was undilated, but enlarged in its whole circumference. Its length was one and a half inches. Thickness of parietes about one inch; very hard and unyielding. When incised longitudinally, it presented a white, firm surface, and yielded a sound like cutting cartilage. The internal surface had begun to ulcerate; hence the fetid discharge for several weeks, which had given us a clue to the character of the disease.

In searching the records of carcinoma uteri, I find no case in which the whole cervix was so uniformly diseased. There are several cases in which one side was affected, and the other dilatable; sufficiently so to allow of the passage of the fœtus *per vias naturales;* but here there was not the slightest yielding. In this case, had the evidence of fœtal life continued during labor, the Cæsarian section would have been justifiable; not so much for any lasting benefit to the mother, as the child; for experience has proved that this terrible disease rapidly advances, after delivery, to a fatal termination.

